# Education in Dental Medicine

**Published:** 1900-04

**Authors:** Harold Williams


### EDUCATION IN DENTAL MEDICINE.[1]

BY HAROLD WILLIAMS, M.D.[2]

If there is any truth in the saying that no one can be more pleasing than one who is clothed in the mantle of modesty, it seems to me that I should meet with a cordial reception at your hands. I am free to confess that when the flattering request was made me that I should address your distinguished association this evening on the subject of Education in Dental Medicine, I was filled with misgivings. It seemed to me presumptuous that I, who am but new at the business, should ventilate my views before you who are in truth a company of experts,—men who have both incidentally and actually given years of time and thought to this important subject. It seemed to me that I was placing myself in the position of the boy who advises his father about the conduct of his business.

But, after all, there are in this subject of Education in Dental Medicine, as in all other questions, two sides; and if I venture to speak to you on the medical side of the subject, perhaps I may not be so arrogant after all. As an outsider, as it were, at your meeting to-night, it is one of my privileges to congratulate you, gentlemen, upon the advance which the dental profession has made in the past fifty years. When we compare the dentist of to-day, performing his

---

[1] Read before the American Academy of Dental Science (Annual Meeting), November 15, 1899.

[2] Dean Tufts College Medical and Dental Schools.

painful operations with the aid of his anæsthetics, or preparing a diseased structure with his modern dental engine and filling its cavity after the approved methods at present in vogue, with the dentist of the previous generation extracting molars from his screaming patient with the key, or driving gold into a tooth-cavity with a mallet and tamp, it indeed seems that not only the profession but the patient as well should be fit subjects of congratulation upon the enormous advances which dentistry has made in the previous half-century. And if these matters alluded to are subjects of felicitation, what shall be said of the enormous strides which dentistry has made in the way of preserving the teeth, of the triumphs of orthodontia, or of the seemingly impossible achievements in the nature of crown- and bridge-work! If the Darwinian theory of evolution be true, we shall find the teeth of the coming generations so regular in position and so perfect in structure that the aid of the dentist will not be requisite. Perhaps, indeed, the process of evolution may be carried to such an extent that we shall see the Arctic explorer and Klondyker of the future walking, like the walrus, over the fields of ice by the aid of his teeth!

But if dentistry has advanced so enormously in the past fifty years, so, also, has the study of medicine. Empirical methods are fast disappearing before the exact knowledge which has come to us through the medium of the microscope and the laboratory, and every year sees the profession of medicine becoming more nearly and more surely an exact science. So rapid, indeed, has been the advance in our knowledge, and so widely has the advancement extended, that it has become necessary to increase the course laid down for the student of medicine from three to four years, and even then it is found that those who direct the education of medical students are put to their trumps to find time to teach their pupils all that it is considered requisite that they should know.

With the recent discoveries in bacteriology, in pathology, in chemistry, in physiology, and in the application of these sciences to medicine and to surgery, vast, indeed, are the advances of the medical sciences during the two decades that are past.

Hitherto, Mother Medicine and her dental step-child have walked together. Side by side they have pursued their way, the child sometimes laggingly, to be sure, in the manner that children are wont to do. Side by side they have prosecuted their efforts for the amelioration of the woes of suffering humanity. But the time is now rapidly bringing them to the parting of the ways, when dentistry is either to be considered as a science,—a special branch, as it were, of medicine,—or when it is to be regarded more in the nature of a highly developed mechanical art. Is it to keep pace with the march of science, or is it to halt and rest by the wayside?

I think I am not departing from the facts of the case when I say that the dental profession of the present day is fast ranging itself in two great bands,—the one which desires to see dentistry practised as an art, and the other which prefers to see it keep step with the modern march of science. In this latter category I desire to enroll myself, and to cast whatever little of influence I may possess. I believe that dentistry should be regarded as a specialty of medicine in a similar manner that the diseases of the eye or the ear are to be so regarded. And it seems to me that the dentist who is called upon to treat a suppurating pulp-cavity must have as complete a knowledge of the nature of the morbid processes which go on in the human body as must the surgeon who is called upon to operate upon a diseased appendix. The disease processes are almost exactly identical in the two cases, differing only in their anatomic relations, and I maintain that no dentist is properly equipped for the practice of his profession whose preliminary training has not qualified him for a true appreciation of the morbid conditions he is called upon to treat. Such knowledge can only come to him from the bacteriological laboratory and the clinic. He must know the life history of the micro-organisms as surely as the surgeon; he must be as thoroughly grounded upon antisepsis and the methods of aseptic surgery as the surgeon himself.

But when I say that I desire to enroll myself among those who believe that dental education should be advanced along the more purely scientific lines of the medical profession, I do not wish to be misunderstood as being one of those advocates who slavishly follow that extension as dictated by some extreme exponents of the doctrine. The present age is the era of election in study, of specialization, and I believe, while extending the line of dental education in the direction of medical science, we should admit of the fullest specialization and individual choice that is consistent with a certain necessary minimum knowledge of the different subjects. Such are my present views. If in the future they should prove to be mistaken, I trust no one will be more liberal than I shall be, or more willing to admit his error.

To consider this matter somewhat further in detail and at the risk of wearing out your patience, I should like to individually allude to the purely scientific branches which enter, at the present time, into the curriculum of a dental school. These branches are anatomy, physiology, chemistry, bacteriology, pathology. Possibly there might also be included theory and practice of general medicine, especially so far as it relates to the etiology and symptomatology of such particular diseases as the dentist is likely to come in contact with. Such a course is now given in our own school, and, it is believed, will prove of value. Taking up these subjects in the order named, general anatomy demands our first consideration. In this study I believe the dental student should receive the same course as that which is laid down for the medical student. The average student in the dental colleges of to-day comes to them with merely a knowledge of books. His only idea or knowledge is book knowledge. His mind is entirely untrained for the study of nature. He has never been taught to learn through the medium of his own special senses. In the first place, therefore, he must be taught how to learn. He must be taught to observe, to discriminate, and to judge, and, having been so taught, he must learn to express this newly acquired knowledge. Above all, he must be taught accuracy, and in anatomy we find a subject most admirably adapted to initiate him into the methods of scientific research. All believe that the dental student should learn the anatomy of the head and neck as an essential to his calling, but without the further knowledge of general anatomy, his knowledge of the head and neck must be, at the best, but incomplete. Much of our knowledge is derived from comparison and analogy, and no person can have a complete knowledge of a part who has not a partial knowledge of the whole. This more complete knowledge of anatomy is, further, to assist him in his comprehension of the physiological problems which he is later to be confronted with; in the physiological phenomena which are, as Harley puts it, the basis upon which the pyramid of medicine is reared. It is also to educate him in the delicate manipulations of dissection and to help him in the education of the fingers, which in the future must become such skilled assistants. But here again the student should be allowed to specialize, and a less exhaustive knowledge of the trunk and extremities should be required of him than is exacted from the student in general medicine. The ideal course in anatomy, it seems to me, should be one in which general anatomy is required of all students, supplemented by elective courses in each of the special branches.

In physiology we are offering the student a course of study which is to give him in a still higher degree the scientific training so necessary for the prosecution of his profession. Not only is he to learn the methods of the various processes which take place during health in the different portions of the body, the applications of chemistry and physical knowledge to the human body, but also is the development of his intellectual faculties to be further carried on. In this branch is his knowledge of the physical sciences to be greatly extended, and in this direction is much of the future advancement of the profession of dentistry to be looked for. In the laboratory method of teaching at present in vogue he is furthermore to educate his mechanical skill by the construction and manipulation of delicate instruments.

In chemistry the student should learn not only those bare facts which are necessary to him in the practice of his calling, but should enter into the outlying fields of physiological and clinical chemistry. He should learn the chemistry of the fluids of the body,—the saliva, gastric juice, and blood,—and of the processes of fermentation, and he should learn enough to enable him to conduct original research and to become enabled to advance that knowledge which he has received from those who have gone before. In the discovery of anæsthesia one of the greatest advancements of the century has come to us through a member of the dental profession. Is it chimerical to hope that with the opportunities at their command some dentist of the future will give us a practical method of local anæsthesia?

Pathology, again, is a study which must be widely extended in the dental curriculum. Not only has the present knowledge of dental pathology been far outdistanced in the advance of science; not only is it absolutely necessary for the dental practitioner to be thoroughly grounded in the laws of general pathology, but also it is needful that he should know something at least of such special pathology as is necessary for him for his own self-protection and the protection of his patients. And if the dentist of to-day were better acquainted with such pathological processes as I have indicated, I venture to assert that the longevity of the profession would be greatly increased.

In bacteriology the student is to learn the life history of those micro-organisms which have come to play so important a part in the study of disease; he is to learn the causes of disease and the method of preventing those causes; he is to learn that in the carious tooth of the child may lurk a poison no less deadly than that which is to be found in the hollow fang of the serpent. In this wonderful study he is to learn the exactitude of science and scientific methods in their highest expression and to prepare himself for the practice of his profession upon its very highest plane. I wonder how many dentists of the present day have ever seen the germs—the underlying causes—of the diseases which they treat. How many of them are familiar with Koch's laws for demonstrating the specific relations between a bacterium and a disease? And if the modern dental student has not studied these causative factors, is it not obvious that he is carrying on his study from the wrong end?

It is only through the study of these scientific branches, it seems to me, that dentistry is to advance, that the intellectual faculties of the dentist of the future are to be so developed that he can offer to his patient the trained judgment of the scientist instead of the rule-o'-thumb empiricism of the mere mechanic. But I do not wish to be here understood to underrate the value of this same mechanical deftness which is essential in the practice of dentistry. On the contrary, no one could be more keenly appreciative than I am of the necessity of such skill or of the extraordinary perfection attained by members of your profession. I have seen work performed by dentists which to me seems simply phenomenal. Almost impossible mechanical achievements have been accomplished by members of your profession, and in admiring the extraordinary mechanical perfection of some of your brethren I have almost wished that man might, like the narwhal, be possessed of teeth nine feet in length and with pulp cavities throughout their entire extent, so marvellous was their handiwork.

Between the mechanical and the scientific lies the golden mean. With the faculties of our dental schools composed of the advocates of the two systems, it seems to me that the bark of dental education may be safely steered. At the same time I believe, with Montaigne, that "Habit is a violent and treacherous schoolmistress," and am convinced that the trend of dental education must be upward and onward, and that four years must be devoted to the study of dental medicine if the American dentist is to maintain his present prestige as the most accomplished in the world.

